# Supplementary material for: Genome-wide identification, characterization and gene expression of BES1 transcription factor family in grapevine (Vitis vinifera L.)
Source: Sci Rep. 2023 Jan 5;13:240. doi: 10.1038/s41598-022-24407-y (PMC9816167; doi:10.1038/s41598-022-24407-y)
Supplement: Supplementary file 3 — Supplementary Information. [file 41598_2022_24407_MOESM3_ESM.zip › Vvi_Atr/Vitis_vinifera.PN40024.v4.dna_sm.toplevel.fa.vs.Amborella_trichopoda.AMTR1.0.dna_sm.toplevel.fa.html/Atr-AmTr_v1.0_scaffold00185.html]

|  |  |  |  |  |  |  |  |  |  |  |  |  |  |
| --- | --- | --- | --- | --- | --- | --- | --- | --- | --- | --- | --- | --- | --- |
| Duplication depth | Reference chromosome | Collinear blocks | | | | | | | | | | | |
| 0 | Atr-ERN14439 |  |  |  |  |  |  |  |  |  |  |  |  |
| 0 | Atr-ERN14440 |  |  |  |  |  |  |  |  |  |  |  |  |
| 0 | Atr-ERN14441 |  |  |  |  |  |  |  |  |  |  |  |  |
| 0 | Atr-ERN14442 |  |  |  |  |  |  |  |  |  |  |  |  |
| 0 | Atr-ERN14443 |  |  |  |  |  |  |  |  |  |  |  |  |
| 1 | Atr-ERN14444 |  | Vvi-Vitvi04g01438\_t001 |  |  |  |  |  |  |  |  |  |  |
| 1 | Atr-ERN14445 |  | \| \| |  |  |  |  |  |  |  |  |  |  |
| 1 | Atr-ERN14446 |  | Vvi-Vitvi04g01437\_t001 |  |  |  |  |  |  |  |  |  |  |
| 2 | Atr-ERN14447 |  | \| \| |  | Vvi-Vitvi18g01083\_t001.1.6037826d |  |  |  |  |  |  |  |  |
| 2 | Atr-ERN14448 |  | \| \| |  | Vvi-Vitvi18g01081\_t001 |  |  |  |  |  |  |  |  |
| 2 | Atr-ERN14449 |  | Vvi-Vitvi04g01436\_t001 |  | \| \| |  |  |  |  |  |  |  |  |
| 2 | Atr-ERN14450 |  | \| \| |  | \| \| |  |  |  |  |  |  |  |  |
| 2 | Atr-ERN14451 |  | Vvi-Vitvi04g02147\_t001 |  | \| \| |  |  |  |  |  |  |  |  |
| 2 | Atr-ERN14452 |  | Vvi-Vitvi04g01434\_t001 |  | \| \| |  |  |  |  |  |  |  |  |
| 2 | Atr-ERN14453 |  | \| \| |  | Vvi-Vitvi18g01080\_t001 |  |  |  |  |  |  |  |  |
| 2 | Atr-ERN14454 |  | \| \| |  | \| \| |  |  |  |  |  |  |  |  |
| 2 | Atr-ERN14455 |  | \| \| |  | Vvi-Vitvi18g01069\_t001 |  |  |  |  |  |  |  |  |
| 2 | Atr-ERN14456 |  | \| \| |  | \| \| |  |  |  |  |  |  |  |  |
| 2 | Atr-ERN14457 |  | \| \| |  | \| \| |  |  |  |  |  |  |  |  |
| 2 | Atr-ERN14458 |  | \| \| |  | Vvi-Vitvi18g01068\_t001 |  |  |  |  |  |  |  |  |
| 2 | Atr-ERN14459 |  | \| \| |  | Vvi-Vitvi18g01067\_t001 |  |  |  |  |  |  |  |  |
| 2 | Atr-ERN14460 |  | \| \| |  | Vvi-Vitvi18g04249\_t001 |  |  |  |  |  |  |  |  |
| 1 | Atr-ERN14461 |  | Vvi-Vitvi04g01432\_t001 |  |  |  |  |  |  |  |  |  |  |
